# Early electrophysiological responses reflect precision-weighted prediction errors to face features

**DOI:** 10.1371/journal.pone.0357956

**Published:** 2026-09-25

**Authors:** Cristiano Costa, Emma K. Ward, Rachele Pezzetta, Fabio Masina, Giorgio Arcara, Clare Press, Cristina Scarpazza

**Affiliations:** 1 Department of General Psychology, Università degli Studi di Padova, Padua, Italy; 2 Department of Experimental Psychology, University College London, London, United Kingdom; 3 IRCCS San Camillo Hospital, Venice, Italy; 4 Functional Imaging Laboratory, University College London, London, United Kingdom; Gabriele d’Annunzio University of Chieti and Pescara: Universita degli Studi Gabriele d’Annunzio Chieti Pescara, ITALY

## Abstract

Facial expressions serve as a key source of information about an individual’s emotional state, intentions, and the surrounding environment. Efficient error processing of facial features is therefore particularly important for successful social interaction. Nonetheless, while simple feature-selective error processing has been well documented, the literature on error processing in complex visual features, such as those of facial stimuli, that may not generate specific predictions at the fine-grained level of simple ones, presents mixed findings. The present study aimed to determine whether facial feature-related processing activity reflects precision-weighted prediction errors (pwPEs), and to investigate how rapidly these feature-selective error signals emerge within the EEG epoch. Using the Hierarchical Gaussian Filter to model pwPE evolution, a consistent relationship was observed between pwPE trajectories and neuronal activity, with peak effects emerging at approximately 140 ms post-stimulus onset. These findings provide evidence that facial expression prediction errors are computed early during face visual processing. During social interactions, this timely error signaling likely enables the continuous updating of predictive models about a social partner’s likely responses to environmental stimuli or interpersonal actions, facilitating rapid and adaptive behaviours that promote smooth social engagement.

## Introduction

As perceptual agents, we are not mere passive recipients of sensory information; instead, our brains actively recapitulate the statistical regularities in the environment and combine these with incoming sensory inputs to represent the likely state of the world [1–3]. This process is argued to be governed by hierarchical predictive coding mechanisms [4,5], where higher-level units generate backward predictions about expected sensory information, which are then compared with the actual sensory input in lower-level units. The resulting prediction errors, which encode the mismatch between the prediction and sensory input, are crucial for updating prior expectations [6,7]. Importantly, prediction error signals are weighted by their precision [8], allowing the brain to prioritize more informative error signals [9].

Perceptual prediction errors are argued to convey not merely a general “surprise” response, but to carry specific representational content [3,10,11]. Several studies employing simple stimuli such as tones or Gabor patches have identified feature-selective error signals, demonstrating that neuronal activity patterns to deviations from expectations contain information about the identity of the predicted stimulus ([12,13] for reviews). For instance, Smout et al. [14] quantified the neuronal selectivity for information related to grating orientation and the mismatch between the predicted and observed orientations, showing that surprising stimuli evoke neuronal responses that contain information about the difference between predicted and observed stimulus features.

However, when it comes to perceiving changes in complex visual features, such as facial expressions, these cannot be effectively translated into predictions at the level of early visual receptive fields that process specific orientations and contrasts in retinotopic space [11]. Correspondingly, studies on error processing related to such complex features present mixed findings, characterized by inconsistent neuronal signatures and inferences that largely rely on canonical latency and peak difference measures [15–19]. These studies are important nonetheless because they suggest that regularities can be abstracted over complex stimuli such as faces, rather than being limited to simple visual dimensions such as orientation or colour. At the same time, they also illustrate a central interpretative difficulty in this literature: differential responses to rare facial expressions may reflect a violation of learned regularities, but may also be influenced by the processing of emotional expression content, structural face encoding, or low-level physical differences between stimulus categories. This has motivated designs in which physically identical faces can be compared under different expectation contexts. Particularly relevant here, Stefanics et al. [20] used a multi-feature (happy, fearful expressions; red, green colour patch) roving-standard paradigm and modelled trial-wise precision-weighted prediction errors (pwPEs). Because stimulus status was determined by local sequence context, the same pictures could serve as expected or unexpected events, providing stronger control over physical stimulus confounds. More recently, work using multivariate fMRI and computational models has further shown that prior expectations can shape face representations across the face processing hierarchy, supporting the view that face perception reflects an interaction between sensory evidence and learned expectations [21]. However, if the error signal does carry representational content, it has yet to be determined how rapidly feature-selective error signals emerge in the context of complex stimuli such as expressions. If this information is timely and perceptual in nature, it would potentially reflect deviations from predicted structural, configural and dynamic aspects of complex stimuli before higher-order cognitive processing occurs [10,22]. If, however, the information is derived at later stages, this would mean that error-related information is only later attributed to particular stimulus features through recurrent processing and integration with higher-order cognition [23]. Indeed, further evidence is needed to understand how sensory predictions and prediction errors are conveyed across processing levels that handle increasingly complex information [3,5,24]. While top-down predictions of more complex attributes may constrain lower-level visual processing as they propagate through the cortical hierarchy [1,25], their downstream transformation may become increasingly diffuse and therefore difficult to detect at earlier stages of the hierarchy [11]. Consistent with this, differential responses to predictable complex images are observed in the ventral temporal cortex, but not in lower visual areas [26,27]. Addressing these questions will help clarify how predictive coding models accommodate complex stimuli that may not generate specific predictions at the fine-grained level of simple features, such as those processed by V1 neurons [28–31], and how these processes support everyday perception and, consequently, behaviour. The present study investigates precision-weighted prediction errors (pwPEs) in response to facial features, which serve as a key example of complex stimuli that cannot easily be reduced to simple spatial predictions, and employs a multi-feature oddball paradigm in which rare changes were presented relative to a common standard. These types of stimuli are a crucial source of information for successfully navigating the world and social interactions, providing cues about an individual’s emotional state and intentions, as well as contextual details about the environment [32,33].

Precisely, the Hierarchical Gaussian Filter (HGF; [34,35]) was employed here to test whether neuronal activity during the processing of facial features covaried with computationally estimated pwPEs estimated under ideal Bayesian observer assumptions, and how rapidly these feature-selective error signals emerge. The HGF is a generative Bayesian model of perceptual inference and learning widely applied to investigate hierarchical prediction error responses in the brain [36–41]. Computational trajectories of pwPEs simulated by the HGF were used as parametric regressors in the electroencephalographic (EEG) data analysis, allowing for a formal, trial-by-trial investigation of the relationship between neuronal responses and these computational quantities.

EEG data for this study were recorded from healthy participants in a multi-feature oddball paradigm. The standard condition featured male faces with neutral expressions, while the deviant condition included three equiprobable types of stimuli: male faces with fearful expressions, male faces with happy expressions, and female faces with neutral expressions. This setup enabled comparisons of neuronal activity across three types of feature-selective deviance detection relative to a common standard. Importantly, the feature of interest had to be automatically extracted from sets of four faces, positioned in the four peripheral quadrants of the monitor, despite variation in low-level visual features. This leveraged the cognitive ability to integrate multiple items and rapidly and precisely extract ensemble statistics from them; a process known as ensemble coding [42–45].

Precision-weighting provides a mechanism through which the brain promotes the influence of error channels that offer the most reliable information for revising sensory expectations [3]. We hypothesised that this mechanism would be expressed rapidly during the processing of facial feature regularities. This prediction was motivated by the fact that faces are highly familiar and behaviourally relevant visual stimuli, for which structural, configural, and expression-related information can be extracted within early stages of visual processing [46–49]. If learned expectations about facial-feature regularities constrain perceptual encoding online, trial-wise EEG activity should covary with HGF-derived pwPEs within the early face-processing epoch. Such a finding would suggest that prediction-error weighting is already operating at the level of perceptual face-feature representations [10,23], rather than only at later decisional or interpretative stages. Conversely, if pwPE-related activity emerged only at later latencies, this would suggest that prediction-error information becomes feature-specific only after further recurrent processing, categorization, attentional orienting, or higher-order model updating [23].

## Materials and methods

### Ethics statement

The study was approved by the ethical committee of the IRCCS San Camillo Hospital (Protocol 2022.10). Data collection began on 2 February 2023 and concluded on 21 April 2023. After an extensive explanation of the experimental procedures, written informed consent was obtained from each participant before the beginning of the experiment, according to the Declaration of Helsinki and its later amendments.

### Participants

A statistical power analysis was conducted using G*Power software [50] to determine the minimum sample size required for the study. The analysis used the F-test family for a repeated-measures ANOVA with a within-participant factor, Cohen’s *f* = 0.25, α =  .05, desired power = .80, one group, three repeated measurements, an assumed correlation among repeated measures of  .50, and a nonsphericity correction ε = 1. This yielded a required sample size of 28 participants. To account for an estimated 10% dropout rate, EEG data were collected from 31 healthy participants recruited from the University of Padua, Italy. We note that this power analysis was not a formal power analysis for the final scalp × time SPM analysis, which used trial-wise computational regressors and cluster-level familywise-error correction. Rather, it was used as an approximate planning criterion in the absence of straightforward power analysis tools for mass-univariate model-based EEG analyses.

Due to technical issues during data acquisition, data from two participants were excluded: one participant’s recording was affected by an amplifier shutdown, and another had one of three experimental blocks that was not recorded. Consequently, the final sample comprised 29 participants (19 females, 10 males; mean age = 25.14 years, *SD* = 2.67; 28 right-handed; education = 17.93 years, *SD* = 1.1). All participants had normal or corrected-to-normal visual acuity and no history of psychiatric or neurological conditions.

### Experimental paradigm and procedure

After providing written informed consent, participants were seated in an armchair in a dimly lit, quiet room. A 27-inch LCD Full-HD (60 Hz refresh rate) monitor was placed at a viewing distance of ∼1 meter. Participants were instructed to remain still, keep quiet, and maintain central eye fixation throughout the task.

A multi-feature visual oddball paradigm was employed to elicit mismatch responses through rare changes in the physical features of human faces, relative to a common standard.

Face pictures of 20 models (10 female, 10 male) were selected from the Chicago Face Database [51] based on the highest prototypicality-to-unusuality ratios. The 10 female models were depicted with a neutral expression, whereas each of the 10 male models was represented with three expressions: neutral, fearful, and mouth-open happy, resulting in a total of 40 pictures.

**Fig 1:**
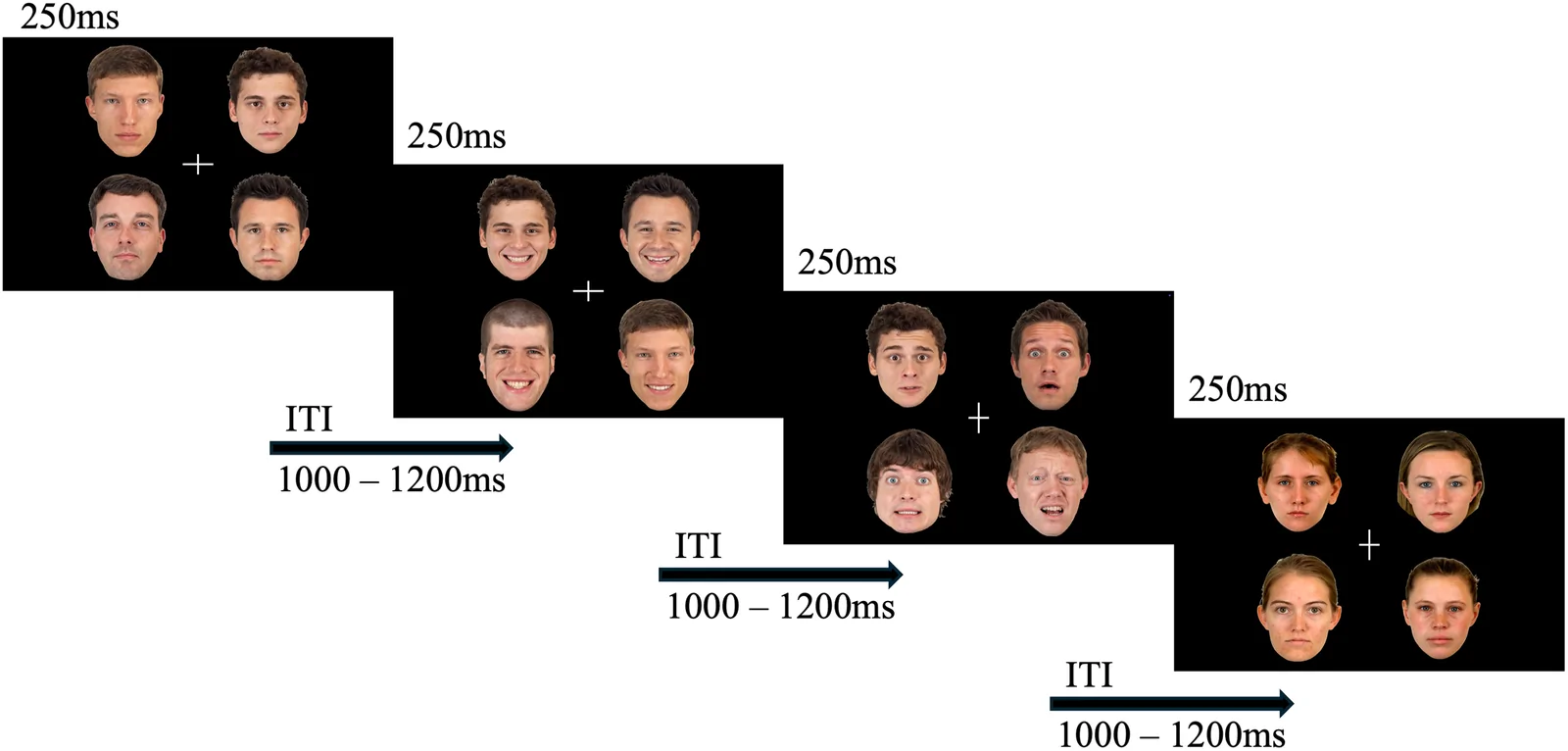
Stimuli and paradigm. Multi-feature visual oddball paradigm to elicit prediction errors by rare changes of human face features. Faces were presented in four peripheral quadrants of the screen. Deviant stimuli (happy and fearful expressions; female neutral expressions) were interspersed with at least three standard stimuli (male neutral expressions). As described in the main text, the length of the white fixation cross varied throughout the task. In this example, in trial 1 and 2, the vertical line is shorter than the horizontal line, while in trial 3 and 4, the horizontal line is shorter than the vertical line. A detection task was presented at fixation at the center. ITI = Intertrial interval.

The experiment was designed and presented with PsychoPy^®^ 3.0.6 [52]. Neutral male faces were presented as frequent standards (85% of trials), while the deviant condition included three equiprobable types of stimuli: faces of the same 10 male models with a fearful expression; faces of the same 10 male models with a happy expression; faces of 10 female models with a neutral expression (each making up 5% of trials). Critically, each stimulus screen (hereafter referred to as a “stimulus”) displayed pictures of four different individuals belonging to the same condition and type, positioned in the four peripheral quadrants of the monitor against a black background (Fig 1).

The presentation order of the pictures within each stimulus was pseudo-randomized, with the restriction that a picture of the same model was not shown twice within the same stimulus or on consecutive stimuli. Deviant stimuli were always interspersed with at least three standard stimuli and were never presented at the beginning or end of a block. Each four-face compound stimulus was displayed for 250 ms, with an intertrial interval (ITI) randomized between 1000 and 1200 ms. A total of 1275 standard and 225 deviant stimuli (75 per type) were presented. Trial order was randomized across participants.

To ensure the stimuli remained task-irrelevant, participants were instructed to detect unpredictable changes in the length of the horizontal and vertical lines of a white fixation cross displayed at the center of the visual field and to respond as quickly as possible to these changes by pressing the spacebar. The use of an incidental task requiring visual fixation aimed to ensure that eye movements and visual search did not confound the evoked responses to the stimuli. Unbeknown to them, they had 500 ms to respond; otherwise, the response was recorded as a miss. The cross occasionally became wider or longer, with changes occurring at a mean frequency of 6 ± 2 per minute. The fixation cross could only change within a time window spanning from 250 ms to 700 ms after the offset of the stimulus, to avoid confounding the ERP response.

Participants initially familiarized with the task through a non-recording training session consisting of 50 trials randomly drawn from the standard condition sample. After confirming their understanding of the instructions, the EEG recording session started. The task was divided into three blocks, each containing 425 standard and 75 deviant (25 per type) stimuli, to allow participants two self-paced breaks. The experiment lasted approximately 35 minutes.

Pictures display actors from the Chicago Face Database [51] and are used in accordance with the corresponding Terms and Conditions.

### EEG recording and preprocessing

Continuous EEG data were recorded using a high-density 128-channel Ag/AgCl active-electrode system (Brain Products, GmbH, Germany) with an ActiCHamp Plus Amplifier (Brain Products, GmbH, Germany). The electrodes were arranged according to the 10−10 system (ActiCap), with the EEG referenced to AFz and the ground electrode placed on Fpz. Electrodes impedance was maintained below 5 kΩ throughout the experiment. A low-pass filter was applied at 100 Hz during recording, and the EEG signal was digitized with 24-bit resolution at a sampling rate of 1000 Hz. A 5-minutes resting state was recorded before the task, though these data are not analyzed further in the present study.

Offline, the EEG data were imported into the EEGLAB toolbox 2023.1 [53] running on Matlab R2023b (The MathWorks, Natick, MA). Continuous data were first downsampled to 500 Hz and filtered within the 0.1–45 Hz frequency band. Data intervals containing extreme peak-to-peak deflections or large bursts of high-frequency activity were identified through visual inspection and subsequently removed. Independent Component Analysis was then performed using the Infomax algorithm [54] to detect and remove artifact components with known time-series and topographies, such as blinks, saccades, and muscle artifacts.

Continuous data were segmented into time-locked epochs extending from 50 ms before to 450 ms after stimulus onset. Epochs were baseline-corrected using the 50 ms pre-stimulus period. Epochs in which button-press responses occurred within the 50 ms pre-stimulus period or during the stimulus presentation were excluded from the analysis. To further reduce potential artifacts, epochs with voltage values exceeding ± 100 µV on any EEG channel were rejected from the analysis. The average number of removed trials from each dataset for the standard condition was 22.31 (*SD* = 12.97), corresponding to 1.75% of the condition’s total trials. For the deviant condition, the mean number of removed trials was as follows: the fearful expression had 1.66 trials removed (*SD* = 1.42), corresponding to 2.21% of the type’s total trials; the happy expression had 1.31 trials removed (*SD* = 1.23), corresponding to 1.75% of the type’s total trials; and the neutral female had 1.38 trials removed (*SD* = 1.27), corresponding to 1.84% of the type’s total trials.

### Precision-weighted prediction errors

The HGF [34,35] was used to estimate computational trajectories of pwPEs and create parametric regressors for a General Linear Model (GLM) analysis. These estimates correspond to trial-wise pwPE quantities that evolve as the observer accumulates evidence about the likelihood of different facial expressions. The resulting pwPE estimates were then used as explanatory variables in a GLM of evoked responses at each peristimulus time point and across channels (see *Scalp* × *time Statistical Parametric Mapping analysis*). In other words, the effect of pwPEs on the amplitude of evoked responses was tested at each point in the scalp × time space.

The HGF consists of two main components: a perceptual model, which describes a hierarchical process for updating beliefs about environmental states based on sensory inputs, and a response model, which generates behavioural responses. Since the employed paradigm did not involve behavioural responses to the stimuli, participant-specific learning parameters could not be estimated from behaviour. We therefore adopted the standard categorical HGF configuration with predefined priors, in line with previous single-trial EEG applications of the HGF [20,41]. This provided a common ideal observer model across participants, while avoiding the estimation of unconstrained individual parameters in the absence of behavioural data. Accordingly, the resulting trajectories should be interpreted as model-based estimates of belief updating under an ideal Bayesian observer model, rather than as individually fitted learning trajectories [35]. Bayes-optimal perceptual parameters were estimated using the MATLAB function *tapas_fitModel* from the HGF toolbox implemented in the freely available open-source software TAPAS (Version 7.1 [55],). Particularly, *tapas_hgf_categorical_config* specified a three-level categorical perceptual model (Level 1: observed categorical outcomes; Level 2: beliefs about outcome probabilities, represented in logit space; Level 3: volatility governing changes in those probabilities), *tapas_bayes_optimal_categorical_config* specified the pseudo-response model, and *tapas_quasinewton_optim_config* specified the optimization algorithm. The estimated trajectories captured the evolution of pwPEs across each trial, peaking when a stimulus represented a change relative to previous stimuli, and subsiding over following repetitions.

Following Stefanics et al. [20] and Weber et al. [41] notations, a simplified explanation of the HGF derivations is now provided. For mathematical details, refer to Mathys et al. [34,35]. The HGF models how hidden states (x) of the world generate sensory inputs (u), and through model inversion, infers these states from sensory data, updating the beliefs across its hierarchical structure. The model assumes that environmental hidden states at all but the first level evolve as a Gaussian random walk, where their variance depends on the state at the next higher level. Since exact Bayesian inversion requires analytically intractable integrations, the HGF uses a quadratic approximation to the variational energies. This approximation provides a set of update equations that allows for efficient trial-by-trial updates of the state variables. Each belief within the model is updated after each trial, resulting in trial-by-trial trajectories of hidden quantities. The update rule in the HGF hierarchy is that at any level i, the update of the posterior mean $\mu_{i}^{\left(k\right)}$of the state $x_{i}$ representing beliefs on trial k is proportional to the precision-weighted prediction error $\varepsilon_{i}^{\left(k\right)}$. This weighted prediction error is the product of the prediction error $\varepsilon_{i-\text{1}}^{\left(k\right)}$ from the level below and a precision ratio $\psi_{i}^{\left(k\right)}$, as follows:


$$\begin{array}{c} \mu_{i}^{\left(k\right)}-\mu_{i}^{\left(k-\text{1}\right)}\propto \psi_{i}^{\left(k\right)}\varepsilon_{i-\text{1}}^{\left(k\right)}=\varepsilon_{i}^{\left(k\right)}\text{.} \end{array}$$ (1)

In the present EEG analysis, the focus was on the precision-weighted prediction error at the second level $\left(\varepsilon_{\text{2}}\right)$, which is crucial for understanding how beliefs about transition probabilities${\;(\mu }_{\text{2}})$ are updated in response to new sensory inputs. This error term is computed by weighting the prediction error at the first level $\left(\mu_{\text{1}}^{\left(k\right)}-s\left(\mu_{\text{2}}^{\left(k-\text{1}\right)}\right)\right)$ by the posterior variance $\sigma_{\text{2}}^{\left(k\right)}$, which determines the effective learning rate (*i.e.*, the precision ratio) at the second level:


$$\begin{array}{c} \mu_{\text{2}}^{\left(k\right)}=\mu_{\text{2}}^{\left(k-\text{1}\right)}+\sigma_{\text{2}}^{\left(k\right)}(\mu_{\text{1}}^{\left(k\right)}-s\left(\mu_{\text{2}}^{\left(k-\text{1}\right)}\right)) \end{array}$$ (2)

where $\mu_{\text{2}}^{\left(k\right)}$ is the updated belief at the second level after trial k; $\mu_{\text{2}}^{\left(k-\text{1}\right)}$denotes the belief at the second level before trial k; $\mu_{\text{1}}^{\left(k\right)}$ is the posterior belief at the first level based on the observed stimulus at time k; and $s\left(\mu_{\text{2}}^{\left(k-\text{1}\right)}\right)$ is the predicted input at the first level based on the belief from the second level.

In the TAPAS [55] implementation of the categorical HGF, trial-wise pwPE trajectories for each participant are returned as a three-dimensional array ($est.traj.epsi\;\in \;R^{trials\times levels\times outcome}$), where each slice correspond to a specific stimulus category. For the present EEG analysis, the second level pwPE associated with the stimulus category presented on each trial was extracted. Formally, for each trial k, the pwPEs was defined as:


$$\begin{array}{c} {pwPE}^{\left(k\right)}=\varepsilon_{\text{2}}^{\left(k\right)}\left(j_{k}\right), \end{array}$$

where $j_{k}$ denotes the observed category on trial k. This yielded one pwPE value per trial, which was aligned with the corresponding single-trial EEG image and used as a parametric regressor in the GLM (see next section).

### Statistical analysis

#### Scalp × time Statistical Parametric Mapping analysis.

Single-trial data were imported into the SPM12 toolbox (https://www.fil.ion.ucl.ac.uk/spm/) and converted into scalp × time images for statistical analysis. The data were interpolated to create a 32 × 32-pixel scalp map for each time point in the post-stimulus interval ranging from 100 ms to 450 ms. The time dimension comprised 176 samples (∼2 ms per sample) in each trial. These images were stacked to form a 3D space-time image volume, which was then smoothed with a Gaussian kernel (Full-Width at Half-Maximum = 16 mm × 16 mm × 16 ms) to accommodate between-subject spatial variability in channel space, following the assumptions of Random Field Theory [56,57]. Entries from trials that had been rejected during EEG preprocessing were removed prior to analysis.

Statistical Parametric Mapping (SPM) across the scalp × time space was performed building a GLM of trial-wise EEG signals. The model incorporated the two stimulus conditions (i.e., standard and deviant) as main regressors, with the three deviant types treated as a single deviant condition for the present analysis. The three deviant types were included to avoid making the task dependent on a single facial feature deviation and to allow multiple rare violations of the same common standard. However, the primary hypothesis did not concern differences between fearful, happy, and female neutral deviants. Rather, it concerned whether trial-wise EEG activity covaried with the magnitude of computationally estimated pwPEs during the processing of facial feature regularities. Therefore, after extracting the pwPE associated with the actually observed category on each trial, the three deviant types were collapsed into a single deviant condition for the SPM analysis. This also avoided estimating separate mass-univariate models for deviant types with relatively few trials per category.

The trial-wise pwPE estimates were included as condition-specific parametric regressors, aligned with the order of single-trial EEG images.

Formally, for each trial k, the GLM can be expressed as:


$$\begin{array}{c} Y^{\left(k\right)}=\beta_{\text{1}}{Std}^{\left(k\right)}+\beta_{\text{2}}{Dev}^{\left(k\right)}+\beta_{\text{3}}({Std}^{\left(k\right)}\times \tilde{{pwPE}^{\left(k\right)}})+\beta_{\text{4}}({Dev}^{\left(k\right)}\times \tilde{{pwPE}^{\left(k\right)}})+\varepsilon^{\left(k\right)}\text{,} \end{array}$$

where $\tilde{{pwPE}^{\left(k\right)}}$ denotes the mean-centered pwPEs. Parametric regressor orthogonalization followed SPM defaults.

Although the HGF model distinguishes four outcome categories, the GLM here implemented collapsed the three deviant categories into a single condition, as outlined before. Consequently, pwPE regressors reflected belief updating about whether the presented stimulus was standard or deviant, irrespective of the specific deviant subtype.

A random-effects group analysis was conducted across all 29 participants using a standard summary statistics approach [58]. One-sample *t*-tests were employed as second-level models separately for each stimulus condition, and *F* tests were used to examine the parametric modulation of trial-wise EEG signals by pwPEs [20,41].

Results that survived familywise error (FWE) correction based on Gaussian Random Field Theory [59] across the entire volume (scalp × time space) at the cluster level (*p* < .05) are reported, with a cluster defining threshold (CDT) of *p* < .001 [60].

#### Event Related Potential (ERP) analysis.

Besides the model-based analysis investigating the covariation of the EEG signal and pwPEs within the two conditions, a cluster-based permutation test across all channels was conducted. This test statistically compared ERPs for each deviant type (i.e., fearful, happy, female neutral) against the standard condition (male neutral), utilizing the FieldTrip-lite [61] algorithm implemented in the EEGLAB toolbox. This post-hoc analysis was not intended to address the primary hypothesis regarding pwPEs, but rather as a sanity check to provide a canonical ERP-based characterization of the data, to ensure that we observed the standard signatures to the presentation of visual events [15–19]. The analysis involved performing a Monte Carlo permutation test, with a *t*-test calculated at each time point and channel to assess differences between conditions. To correct for multiple comparisons and account for the spatiotemporal correlations in the data, a cluster-based correction was applied [62]. Clusters of significant differences were identified across time points and neighboring channels, with spatial relationships determined using the ‘triangulation’ method. The initial significance level for the *t*-tests was set at *p* = .05, and 4000 permutations were conducted to generate a null distribution of cluster statistics, which allowed for the assessment of the significance of the observed clusters. Clusters were defined using the ‘maxsum’ statistic, which sums the *t*-values within connected components to identify the most prominent clusters of significant activity.

## Results

### Scalp × time Statistical Parametric Mapping analysis

**Fig 2:**
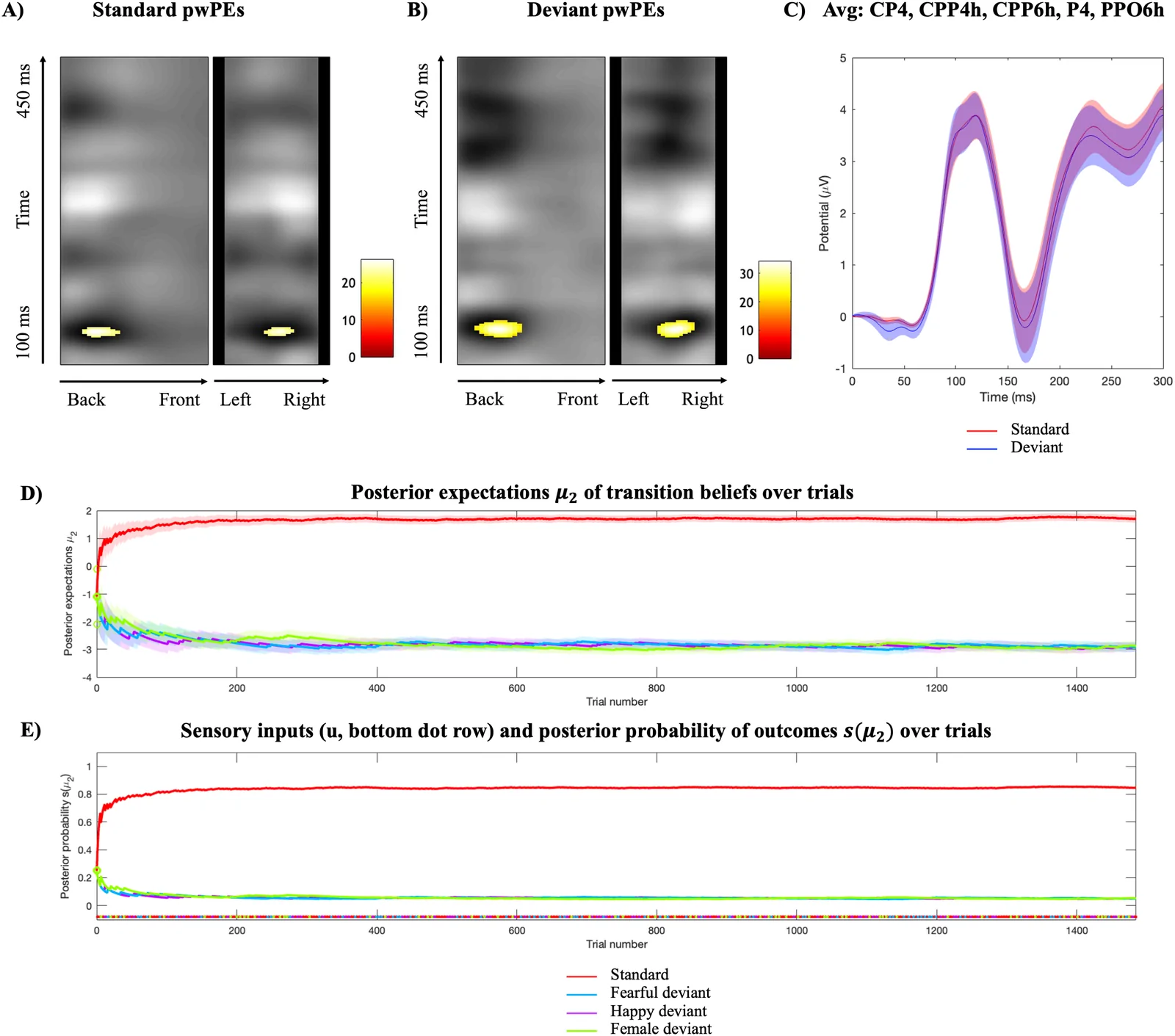
*Top panel:* Thresholded scalp × time Statistical Parametric Maps (SPM) shown as maximum-intensity projections from two views (Back-to-Front, Left-to-Right). The coloured regions indicate scalp × time clusters surviving whole-volume cluster-level FWE correction at *p* < .05 (cluster-defining threshold: *p* < .001). A) Parametric effect of trial-wise standard pwPE estimates on single-trial EEG amplitudes (*F* test). B) Parametric effect of trial-wise deviant pwPE estimates on single-trial EEG amplitudes (*F* test). C) ERP waveforms for the deviant and standard conditions averaged across channels within the significant clusters (CP4, CPP4h, CPP6h, P4, PPO6h). Shaded areas represent Standard Errors. Avg = Average. *Bottom panel:* Example HGF trajectories from one representative participant (ID_01). D) Posterior expectations $\mu_{\text{2}}$ of tendencies $x_{\text{2}}$, showing the evolution of second-level beliefs for the standard and three deviant categories. E) Posterior probability of outcomes $s(\mu_{\text{2}})$; the coloured dots along the bottom indicate the actual stimulus category presented on each trial.

**Fig 3:**
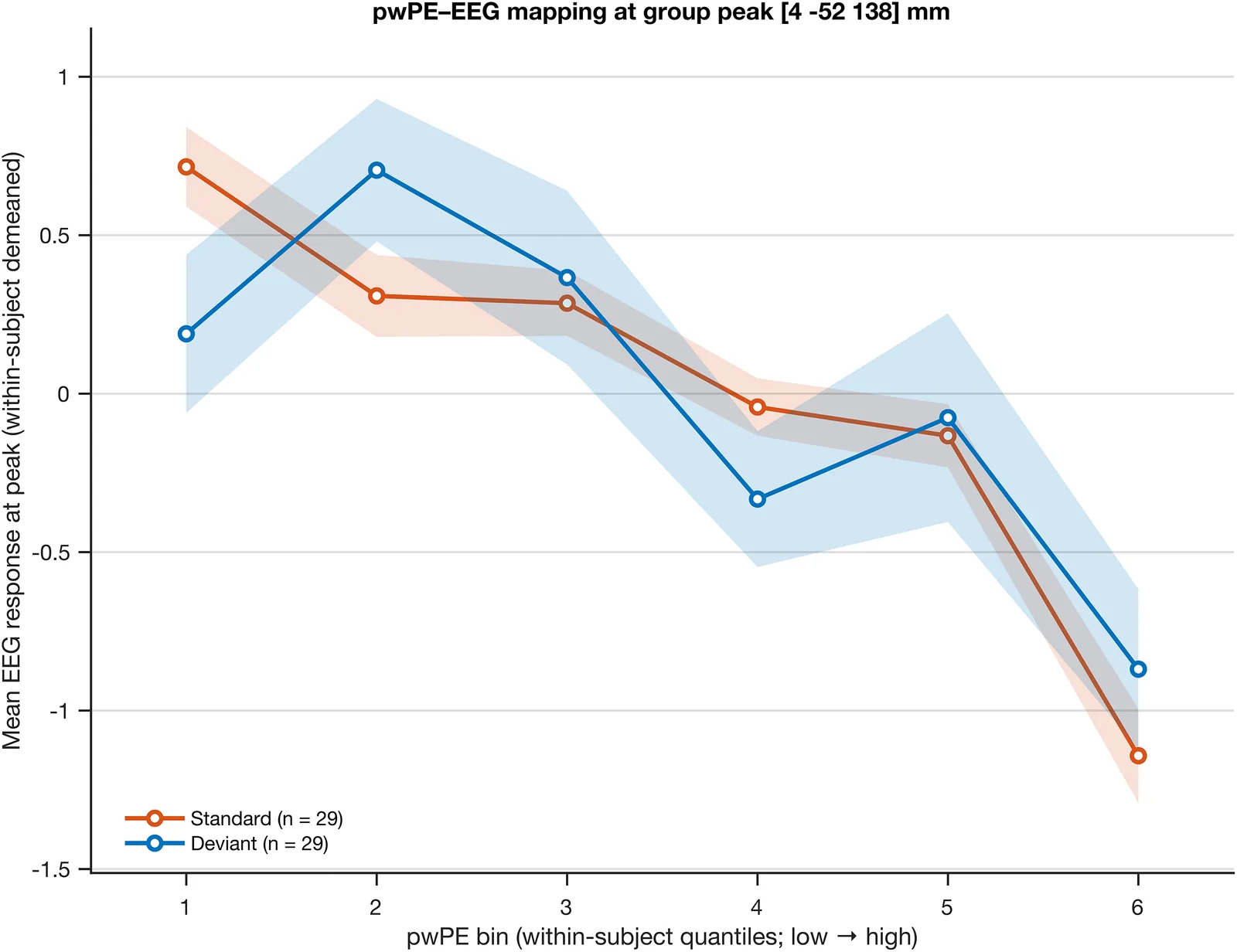
Trial-wise relationship between precision-weighted prediction errors (pwPEs) and EEG amplitudes at the peak of the group-level effect. For visualisation purposes, trial-wise pwPE values were divided into within-participant quantile bins separately for the standard and deviant conditions. Single-trial EEG amplitudes were then extracted at the group-level peak voxel ([4 −52 138] mm), demeaned within participant, and averaged within each bin. Points represent group means across participants and error bars denote the Standard Error. The figure illustrates the parametric relationship formally tested in the scalp × time GLM, showing that larger pwPEs were associated with more negative EEG amplitudes for both standard and deviant stimuli.

By fitting computational trajectories to participants’ single-trial EEG data, a significant trial-by-trial relationship between $\varepsilon_{\text{2}}$ (the pwPEs) and EEG activity was identified for both the standard and deviant conditions. Particularly, for the standard condition, this relationship peaked at 138 ms at middle parieto-occipital channels (peak, $F_{(\text{1},\;\text{2}\text{8})\;}$= 26.08; whole-volume cluster-level FWE corrected, *p* = .031; Fig 2A). For the deviant condition, the relationship peaked at 140 ms at middle parieto-occipital channels (peak, $F_{\left(\text{1},\;\text{2}\text{8}\right)}\;$= 34; whole-volume cluster-level FWE corrected, *p* = .008; Fig 2B). Fig 3 shows the mean single-trial EEG amplitude at the peak of the group-level effect as a function of within-participant pwPE quantile bins for the standard and deviant conditions. Larger pwPEs were associated with progressively more negative EEG amplitudes at the peak of the group-level effect for both the standard and deviant conditions.

A *t*-contrast was run to directly compare the statistical maps for deviant pwPEs and standard pwPEs, which did not yield any significant clusters at the whole-volume FWE-corrected level, suggesting that the relationship between pwPEs and EEG activity are similarly distributed across the scalp × time space for both conditions.

Fig 2C shows the ERPs for the deviant and standard conditions, averaged across channels located within the significant clusters obtained in the scalp × time SPM analysis.

Panels D and E illustrate the rapid stabilization of beliefs under the learned stimulus statistics, with the standard category becoming highly probable and the three deviant categories remaining comparatively unlikely. Particularly, panel D shows the agent’s evolving beliefs about input likelihoods based on past observations, whereas panel E reflects the estimated likelihood of encountering specific stimuli.

### Event Related Potential analysis

**Fig 4:**
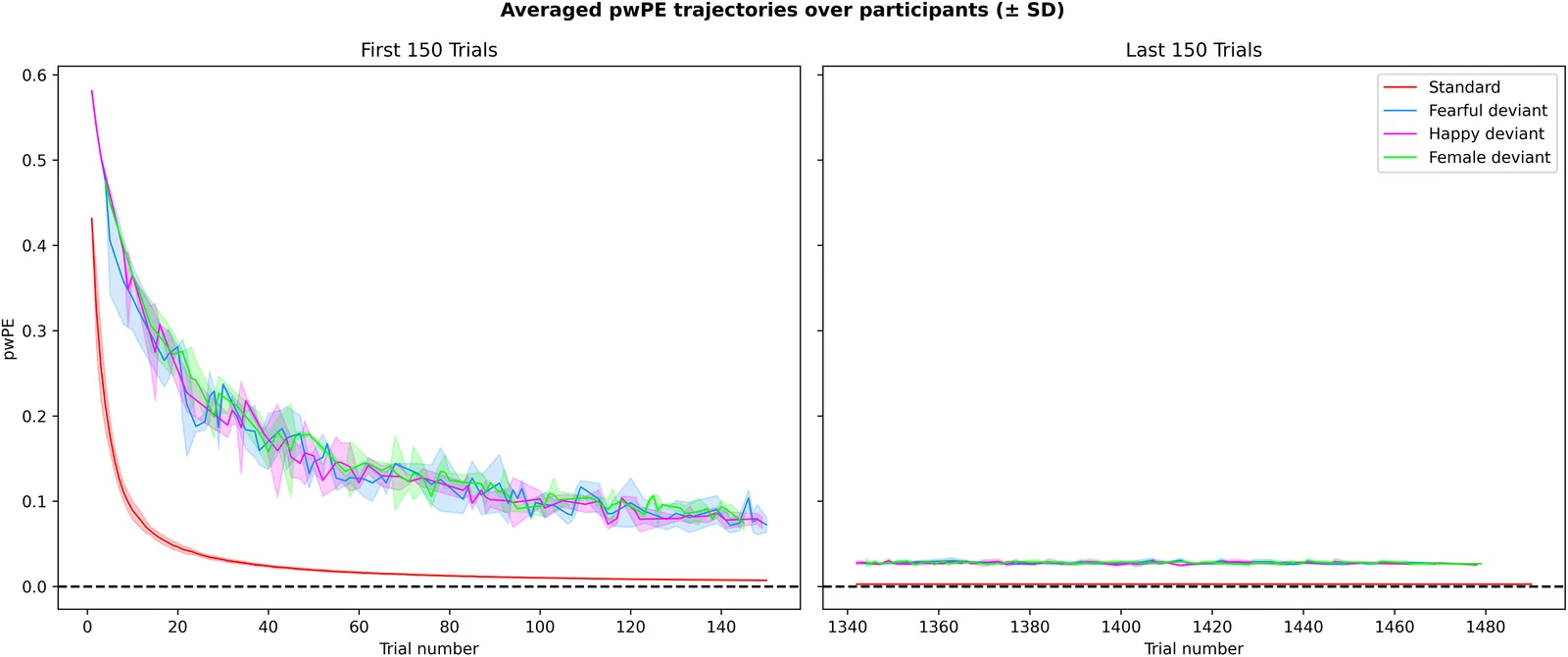
Averaged pwPE trajectories across participants. The two snapshots (left: first 150 trials; right: last 150 trials) show the average evolution of pwPEs over trials, peaking when a stimulus represented a change relative to preceeding stimuli, and subsiding with subsequent repetitions. Shaded colors represent Standard Errors.

The cluster-based permutation test across all channels, conducted to statistically compare ERPs between each deviant type and the standard condition, did not yield any statistically significant difference between the three deviant types and the standard condition. This may be explained by the system’s rapid adaptation to the statistical structure of the stimuli, as suggested by the HGF model estimates (Fig 2D and 2E; Fig 4), leading to reduced responsiveness to deviant stimuli (see Discussion section).

### Reaction time and hit rate

Reaction times and hit rates for the occasional changes in the fixation cross were calculated in the R statistical environment. Data handling and analysis were performed using the “dplyr” [63], “tidyr” [64] and “stringr” [65] packages. The mean reaction time was 470 ms (*SD* = 107 ms), and the mean hit rate was 89.9% (*SD* = 5.63; range: 70.1–98.6%).

## Discussion

The present study aimed to determine whether neuronal activity during the processing of facial features covaried with computationally estimated precision-weighted prediction errors (pwPEs), where precision encodes the reliability afforded by such errors, and investigate how rapidly these feature-selective error signals emerge within the EEG epoch.

To achieve this, the HGF [34,35] was employed to implement a surprise-minimizing Bayesian observer model that simulated computational trajectories of pwPEs related to the conditional transition probabilities of visual outcomes $\left(\varepsilon_{\text{2}}\right)$. These trajectories were incorporated as parametric regressors in a General Linear Model analysis of EEG data.

The results revealed a consistent relationship between pwPE trajectories and neuronal activity, with peak effects emerging at approximately 140 ms post-stimulus onset at midline parieto-occipital channels in the deviant condition. A comparable effect was observed in the standard condition, peaking around 138 ms, providing evidence that neuronal activity reflects pwPE evolution during early face processing stages. Provided that a core function of the human brain is to use sensory representations to predict future states and adapt behaviours accordingly [7,66], rapid and accurate error processing to face features visual inputs may prove particularly important, facilitating timely adaptive behaviours [45,67].

These findings align with the hierarchical nature of visual processing [68,69], wherein sensory areas first process perceptual information while being subject to top-down predictive modulation. Prediction errors are propagated up the hierarchy to update higher-level representations that, in turn, refine top-down predictions [3–5]. As visual information ascends the processing hierarchy, higher-order areas likely encode complex stimulus attributes [70,71] and contextual dependencies [70]. Accordingly, the current results support and extend literature showing that predictive coding mechanisms extend beyond low-level visual receptive fields [3,30,31] to encompass higher-level vision [10,27] and, particularly, the processing of feature-selective deviations from expectations in socially relevant stimuli, such as facial expressions [21]. Moreover, the present study provides evidence for a rapid and efficient reconciliation of sensory input with top-down predictions, corroborating prior research showing that early neuronal responses index perceptual prediction and error resolution in the visual domain [20].

Faces are indeed socially relevant stimuli, but the present task did not require explicit social judgements. Participants were not asked to judge expression, sex, identity, or interpersonal meaning; instead, they performed an incidental fixation task while faces were task-irrelevant. The present findings should therefore be interpreted as evidence that early EEG activity covaries with HGF-derived pwPEs during the processing of facial-feature regularities. This suggests that learned expectations may constrain perceptual encoding online, with prediction-error weighting operating at the level of perceptual face-feature representations [10,23], rather than providing direct evidence for social inference.

Nevertheless, the social relevance of faces provides an important motivation for the present study. In the context of social interactions, timely error signaling likely contributes to the continuous updating of predictive models regarding a social partner’s likely responses to environmental stimuli or interpersonal actions. This process enables adaptive behavioural adjustments that promote smooth interactions and social harmony. Social partners can be conceptualized as dynamic systems with states that fluctuate over time [71]. If one has an internal model of another’s behaviour, accurate prediction of their response to specific environmental or interpersonal inputs should be possible [11]. Rapidly detecting discrepancies between predicted and actual expressions facilitates adaptive social exchanges by enabling efficient and appropriate behavioural adjustments [71]. The presence of early pwPE signatures is also consistent with evidence that the human visual system extracts information from faces within a few hundred milliseconds [46] and even in the absence of conscious awareness [47]. In fact, humans are highly specialized for facial perception [48], and our visual system is refined through experience to detect subtle variations in socially significant facial gestures [49]. Additional insight into predictive coding mechanisms in facial expression perception and the importance of timely error signaling comes from the sensorimotor simulation framework [49], which suggests that emotion recognition can be facilitated by sensorimotor simulation. Beyond visual and contextual processing routes, individuals may partially recreate the motor patterns of observed facial expressions, generating subthreshold motor activity that activates neural systems associated with producing the corresponding emotion [49,72,73]. The observation of early pwPEs may tentatively suggest that predictive error signaling not only updates perceptual representations but also contribute to the rapid recalibration of motor-affective expectations, allowing for timely adjustments in the prediction of another individual’s likely emotional state or subsequent behaviour [49,71].

It is important to note that the experimental design may not have maximized the surprise elicited by deviant stimuli. Surprise is computationally operationalized as the “Kullback-Leibler divergence”, a measure that quantifies the change between beliefs about environmental states before and after sensory evidence [9]. This is suggested by the HGF model estimates (Fig 2, bottom panel), which indicate that a Bayesian observer minimizing surprise would rapidly learn the statistical structure of the task. This is evident in the quickly stabilizing posterior expectations $\mu_{\text{2}}$ of tendencies $x_{\text{2}}$ (Fig 2D), which represent the agent’s evolving beliefs about input likelihoods based on past observations. Similarly, the posterior probability of outcomes $s(\mu_{\text{2}})$ (Fig 2E), which reflects the estimated likelihood of encountering specific stimuli, converged rapidly toward the actual event probabilities. Consequently, the neural system’s adaptation to the task structure likely resulted in reduced responsiveness to deviant stimuli, potentially contributing to the lack of statistically significant differences in the cluster-based permutation test comparing ERP conditions. Nevertheless, one might reasonably anticipate a visual mismatch negativity (vMMN) or differences at the level of the P3 component. However, the literature on complex visual features, particularly facial expressions, has yielded mixed findings. Some studies report a “negativity bias”, with vMMN amplitudes showing larger responses and/or earlier onsets for fearful compared to happy faces [17,18]. In contrast, others describe a “positivity advantage”, whereby happy expressions elicit earlier vMMN onsets than sad or fearful expressions [16,19]. When neutral expressions have been used as standards and emotional expressions as deviants, studies have often found oddball-standard differences without a clear distinction between positive and negative expression [15,74]. Akin to the present study, Stefanics et al. [20], using a roving-standard version of the present paradigm, reported no “canonical” vMMN effects between deviant and standard expressions when analysed with conventional ERP methods.

Lastly, given the relatively modest sample size and the fact that the original power calculation only approximated the final analyses carried out in the present paper, the absence of significant vMMN effects or other deviant-standard ERP differences should be interpreted cautiously, as these null effects may reflect limited statistical power rather than the absence of categorical mismatch responses.

Overall, the present study underscores the explanatory role of predictive coding in facial expression perception, demonstrating that neuronal responses to facial expressions are contingent on the alignment between ongoing predictions and sensory input. These findings reinforce the view that perceptual processing is governed by dynamic adjustments to internal predictive models rather than simple bottom-up sensory evidence [6,10,75]. Additionally, this study highlights the value of integrating computational modeling approaches, such as the HGF [34,35], with traditional ERP analysis. This methodological synergy enhances the detection of subtle neural dynamics that might otherwise be overlooked, offering novel, model-based insights into the neural mechanisms underlying visual processing and emotional expression perception.

### Limitations

This study is not devoid of limitations regarding the interpretation of the findings. First, because the task did not require behavioural responses to the stimuli, individual Bayesian belief updating processes were inferred through a surprise-minimizing optimized Bayesian observer model (e.g.[20,41],) rather than directly derived from the data.

Secondly, the absence of eye-tracking data prevents us from being certain that participants consistently maintained fixation on the central task and did not shift their gaze toward the peripheral face stimuli. Although the hit rate for the speeded button-press responses was high, this does not entirely preclude the possibility that participants may have occasionally shifted their attention to the face stimuli. This could have increased the precision of the sensory input, leading to a corresponding increase in the amplitude of the evoked response [36,76].

Lastly, while using a standard condition common to all deviant types, rather than comparing deviant and standard responses to physically identical stimuli, offers the advantage of directly comparing different features against a consistent baseline, it also introduces challenges. Specifically, the low-level physical differences between the stimuli may have influenced evoked responses, even within the context of ensemble coding, where the brain is thought to extract the mean common feature across a set of stimuli. These low-level variations could potentially confound the interpretation of the results, making it difficult to disentangle the effects of feature-selective deviance detection from those driven by basic physical differences.

To address these limitations and further clarify the findings, future studies should rely on preregistered protocols and incorporate larger sample sizes, more fine-grained tasks, such as implementing more volatile environments to enhance the variability of pwPEs, and utilize stimuli with clearer dimensional differences to increase the likelihood of observing distinct neuronal signals.

## Conclusions

The present study investigated whether neuronal activity during the processing of facial features covaried with computationally estimated precision-weighted prediction errors (pwPEs), and how rapidly these feature-selective error signals emerge.

Using the Hierarchical Gaussian Filter to model pwPE trajectories, a consistent relationship was observed between pwPE trajectories and neuronal activity, with peak effects emerging at approximately 140 ms post-stimulus onset. These findings provide evidence that facial expression prediction errors are computed early during face visual processing. During social interactions, this timely error signaling likely enables the continuous updating of predictive models about a social partner’s likely responses to environmental stimuli or interpersonal actions, facilitating rapid and adaptive behavioural adjustments that promote smooth social engagement.

## References

[1] Clark A. Whatever next? Predictive brains, situated agents, and the future of cognitive science. Behav Brain Sci. 2013;36(3):181–204. doi: 10.1017/S0140525X12000477 23663408

[2] Clark A. Surfing uncertainty: Prediction, action, and the embodied mind [Internet]. Oxford University Press; 2015 [cited 2026 Jul 17]. Available from: https://books.google.com/books?hl=it&lr=&id=TnqECgAAQBAJ&oi=fnd&pg=PP1&dq=Clark,+A.+(2015).+Surfing+uncertainty:+Prediction,+action,+and+the+embodied+mind.+Oxford+University+Press.&ots=avzj4mBaIQ&sig=wJOzh69WZx10_UQjLErDzUbbHSk

[3] Walsh KS, McGovern DP, Clark A, O’Connell RG. Evaluating the neurophysiological evidence for predictive processing as a model of perception. Ann N Y Acad Sci. 2020;1464(1):242–68. doi: 10.1111/nyas.14321 32147856 PMC7187369

[4] Bastos AM, Usrey WM, Adams RA, Mangun GR, Fries P, Friston KJ. Canonical microcircuits for predictive coding. Neuron. 2012;76(4):695–711. doi: 10.1016/j.neuron.2012.10.038 23177956 PMC3777738

[5] Keller GB, Mrsic-Flogel TD. Predictive Processing: A Canonical Cortical Computation. Neuron. 2018 Oct 24;100(2):424–35. doi: 10.1016/j.neuron.2018.10.003 30359606 PMC6400266

[6] de Lange FP, Heilbron M, Kok P. How Do Expectations Shape Perception? Trends Cogn Sci. 2018;22(9):764–79. doi: 10.1016/j.tics.2018.06.002 30122170

[7] Friston K. A theory of cortical responses. Philos Trans R Soc Lond B Biol Sci. 2005;360(1456):815–36. doi: 10.1098/rstb.2005.1622 15937014 PMC1569488

[8] Feldman H, Friston K. Attention, Uncertainty, and Free-Energy. Front Hum Neurosci. 2010;4. doi: 10.3389/fnhum.2010.00215 PMC3001758 21160551

[9] Press C, Kok P, Yon D. The Perceptual Prediction Paradox. Trends Cogn Sci. 2020;24(1):13–24. doi: 10.1016/j.tics.2019.11.003 31787500

[10] den Ouden HEM, Kok P, de Lange FP. How prediction errors shape perception, attention, and motivation. Front Psychol. 2012;3:548. doi: 10.3389/fpsyg.2012.00548 23248610 PMC3518876

[11] Koster-Hale J, Saxe R. Theory of mind: a neural prediction problem. Neuron. 2013;79(5):836–48. doi: 10.1016/j.neuron.2013.08.020 24012000 PMC4041537

[12] Braga A, Schönwiesner M. Neural Substrates and Models of Omission Responses and Predictive Processes. Front Neural Circuits. 2022;16:799581. doi: 10.3389/fncir.2022.799581 35177967 PMC8844463

[13] Heilbron M, Chait M. Great Expectations: Is there Evidence for Predictive Coding in Auditory Cortex?. Neuroscience. 2018;389:54–73. doi: 10.1016/j.neuroscience.2017.07.061 28782642

[14] Smout CA, Tang MF, Garrido MI, Mattingley JB. Attention promotes the neural encoding of prediction errors. PLoS Biol. 2019;17(2):e2006812. doi: 10.1371/journal.pbio.2006812 30811381 PMC6411367

[15] Astikainen P, Cong F, Ristaniemi T, Hietanen JK. Event-related potentials to unattended changes in facial expressions: detection of regularity violations or encoding of emotions? Front Hum Neurosci. 2013;7:557. doi: 10.3389/fnhum.2013.00557 24062661 PMC3769632

[16] Csukly G, Stefanics G, Komlósi S, Czigler I, Czobor P. Emotion-related visual mismatch responses in schizophrenia: impairments and correlations with emotion recognition. PLoS One. 2013;8(10):e75444. doi: 10.1371/journal.pone.0075444 24116046 PMC3792116

[17] Kimura M, Kondo H, Ohira H, Schröger E. Unintentional temporal context-based prediction of emotional faces: an electrophysiological study. Cereb Cortex. 2012;22(8):1774–85. doi: 10.1093/cercor/bhr244 21945904

[18] Stefanics G, Csukly G, Komlósi S, Czobor P, Czigler I. Processing of unattended facial emotions: a visual mismatch negativity study. Neuroimage. 2012;59(3):3042–9. doi: 10.1016/j.neuroimage.2011.10.041 22037000

[19] Zhao L, Li J. Visual mismatch negativity elicited by facial expressions under non-attentional condition. Neurosci Lett. 2006;410(2):126–31. doi: 10.1016/j.neulet.2006.09.081 17081690

[20] Stefanics G, Heinzle J, Horváth AA, Stephan KE. Visual Mismatch and Predictive Coding: A Computational Single-Trial ERP Study. J Neurosci. 2018;38(16):4020–30. doi: 10.1523/JNEUROSCI.3365-17.2018 29581379 PMC6705923

[21] Garlichs A, Blank H. Prediction error processing and sharpening of expected information across the face-processing hierarchy. Nat Commun. 2024;15(1):3407. doi: 10.1038/s41467-024-47749-9 38649694 PMC11035707

[22] Meyer T, Olson CR. Statistical learning of visual transitions in monkey inferotemporal cortex. Proc Natl Acad Sci. 2011;108(48):19401–6. doi: 10.1073/pnas.1112895108 PMC3228439 22084090

[23] Peelen MV, Kastner S. A neural basis for real-world visual search in human occipitotemporal cortex. Proc Natl Acad Sci. 2011;108(29):12125–30. doi: 10.1073/pnas.1101042108 PMC3141945 21730192

[24] Walsh KS, McGovern DP. Expectation Suppression Dampens Sensory Representations of Predicted Stimuli. J Neurosci. 2018;38(50):10592–4. doi: 10.1523/JNEUROSCI.2133-18.2018 30541769 PMC6580665

[25] Rao RP, Ballard DH. Predictive coding in the visual cortex: a functional interpretation of some extra-classical receptive-field effects. Nat Neurosci. 1999;2(1):79–87. doi: 10.1038/4580 10195184

[26] den Ouden HEM, Daunizeau J, Roiser J, Friston KJ, Stephan KE. Striatal prediction error modulates cortical coupling. J Neurosci. 2010;30(9):3210–9. doi: 10.1523/JNEUROSCI.4458-09.2010 20203180 PMC3044875

[27] Egner T, Monti JM, Summerfield C. Expectation and surprise determine neural population responses in the ventral visual stream. J Neurosci. 2010;30(49):16601–8. doi: 10.1523/JNEUROSCI.2770-10.2010 21147999 PMC3975573

[28] Kok P, Jehee JFM, de Lange FP. Less is more: expectation sharpens representations in the primary visual cortex. Neuron. 2012;75(2):265–70. doi: 10.1016/j.neuron.2012.04.034 22841311

[29] Kok P, van Lieshout LLF, de Lange FP. Local expectation violations result in global activity gain in primary visual cortex. Sci Rep. 2016;6:37706. doi: 10.1038/srep37706 27874098 PMC5118700

[30] Richter D, Ekman M, de Lange FP. Suppressed Sensory Response to Predictable Object Stimuli throughout the Ventral Visual Stream. J Neurosci. 2018;38(34):7452–61. doi: 10.1523/JNEUROSCI.3421-17.2018 30030402 PMC6596138

[31] Utzerath C, St John-Saaltink E, Buitelaar J, de Lange FP. Repetition suppression to objects is modulated by stimulus-specific expectations. Sci Rep. 2017;7(1):8781. doi: 10.1038/s41598-017-09374-z 28821808 PMC5562860

[32] Kroczek LOH, Mühlberger A. Neural mechanisms underlying the interactive exchange of facial emotional expressions. Soc Cogn Affect Neurosci. 2025;20(1):nsaf001. doi: 10.1093/scan/nsaf001 39821290 PMC11781275

[33] Wieser MJ, Brosch T. Faces in context: a review and systematization of contextual influences on affective face processing. Front Psychol. 2012;3:471. doi: 10.3389/fpsyg.2012.00471 23130011 PMC3487423

[34] Mathys CD, Lomakina EI, Daunizeau J, Iglesias S, Brodersen KH, Friston KJ, et al. Uncertainty in perception and the Hierarchical Gaussian Filter. Front Hum Neurosci. 2014;8:825. doi: 10.3389/fnhum.2014.00825 25477800 PMC4237059

[35] Mathys C, Daunizeau J, Friston KJ, Stephan KE. A bayesian foundation for individual learning under uncertainty. Front Hum Neurosci. 2011;5:39. doi: 10.3389/fnhum.2011.00039 21629826 PMC3096853

[36] Stefanics G, Kremláček J, Czigler I. Visual mismatch negativity: a predictive coding view. Front Hum Neurosci. 2014;8:666. doi: 10.3389/fnhum.2014.00666 25278859 PMC4165279

[37] Auksztulewicz R, Friston KJ, Nobre AC. Task relevance modulates the behavioural and neural effects of sensory predictions. PLoS Biol. 2017;15(12):e2003143. doi: 10.1371/journal.pbio.2003143 29206225 PMC5730187

[38] Diaconescu AO, Mathys C, Weber LAE, Daunizeau J, Kasper L, Lomakina EI, et al. Inferring on the intentions of others by hierarchical Bayesian learning. PLoS Comput Biol. 2014;10(9):e1003810. doi: 10.1371/journal.pcbi.1003810 25187943 PMC4154656

[39] Diaconescu AO, Mathys C, Weber LAE, Kasper L, Mauer J, Stephan KE. Hierarchical prediction errors in midbrain and septum during social learning. Soc Cogn Affect Neurosci. 2017;12(4):618–34. doi: 10.1093/scan/nsw171 28119508 PMC5390746

[40] Iglesias S, Mathys C, Brodersen KH, Kasper L, Piccirelli M, den Ouden HEM, et al. Hierarchical prediction errors in midbrain and basal forebrain during sensory learning. Neuron. 2013;80(2):519–30. doi: 10.1016/j.neuron.2013.09.009 24139048

[41] Weber LA, Diaconescu AO, Mathys C, Schmidt A, Kometer M, Vollenweider F, et al. Ketamine Affects Prediction Errors about Statistical Regularities: A Computational Single-Trial Analysis of the Mismatch Negativity. J Neurosci. 2020;40(29):5658–68. doi: 10.1523/JNEUROSCI.3069-19.2020 32561673 PMC7363468

[42] Alvarez GA. Representing multiple objects as an ensemble enhances visual cognition. Trends Cogn Sci. 2011;15(3):122–31. doi: 10.1016/j.tics.2011.01.003 21292539

[43] Haberman J, Whitney D. Rapid extraction of mean emotion and gender from sets of faces. Curr Biol. 2007;17(17):R751-3. doi: 10.1016/j.cub.2007.06.039 17803921 PMC3849410

[44] Haberman J, Whitney D. Seeing the mean: ensemble coding for sets of faces. J Exp Psychol Hum Percept Perform. 2009;35(3):718–34. doi: 10.1037/a0013899 19485687 PMC2696629

[45] Ji L, Chen Z, Zeng X, Sun B, Fu S. Automatic processing of unattended mean emotion: Evidence from visual mismatch responses. Neuropsychologia. 2024;202:108963. doi: 10.1016/j.neuropsychologia.2024.108963 39069120

[46] Bijlstra G, Holland RW, Wigboldus DH. The social face of emotion recognition: Evaluations versus stereotypes. J Exp Soc Psychol. 2010;46(4):657–63.

[47] Tamietto M, Castelli L, Vighetti S, Perozzo P, Geminiani G, Weiskrantz L, et al. Unseen facial and bodily expressions trigger fast emotional reactions. Proc Natl Acad Sci. 2009;106(42):17661–6. doi: 10.1073/pnas.0908994106 PMC2764895 19805044

[48] Haxby J, Hoffman E, Gobbini M. The distributed human neural system for face perception. Trends Cogn Sci. 2000;4(6):223–33. doi: 10.1016/s1364-6613(00)01482-0 10827445

[49] Wood A, Rychlowska M, Korb S, Niedenthal P. Fashioning the Face: Sensorimotor Simulation Contributes to Facial Expression Recognition. Trends Cogn Sci. 2016;20(3):227–40. doi: 10.1016/j.tics.2015.12.010 26876363

[50] Faul F, Erdfelder E, Lang A-G, Buchner A. G*Power 3: a flexible statistical power analysis program for the social, behavioral, and biomedical sciences. Behav Res Methods. 2007;39(2):175–91. doi: 10.3758/bf03193146 17695343

[51] Ma DS, Correll J, Wittenbrink B. The Chicago face database: A free stimulus set of faces and norming data. Behav Res Methods. 2015;47(4):1122–35. doi: 10.3758/s13428-014-0532-5 25582810

[52] Peirce J, Gray JR, Simpson S, MacAskill M, Höchenberger R, Sogo H, et al. PsychoPy2: Experiments in behavior made easy. Behav Res Methods. 2019;51(1):195–203. doi: 10.3758/s13428-018-01193-y 30734206 PMC6420413

[53] Delorme A, Makeig S. EEGLAB: an open source toolbox for analysis of single-trial EEG dynamics including independent component analysis. J Neurosci Methods. 2004;134(1):9–21. doi: 10.1016/j.jneumeth.2003.10.009 15102499

[54] Makeig S, Bell A, Jung TP, Sejnowski TJ. Independent Component Analysis of Electroencephalographic Data. In: Advances in Neural Information Processing Systems [Internet]. MIT Press; 1995 [cited 2025 Apr 23]. Available from: https://proceedings.neurips.cc/paper_files/paper/1995/hash/754dda4b1ba34c6fa89716b85d68532b-Abstract.html

[55] Frässle S, Aponte EA, Bollmann S, Brodersen KH, Do CT, Harrison OK, et al. TAPAS: An Open-Source Software Package for Translational Neuromodeling and Computational Psychiatry. Front Psychiatry. 2021;12:680811. doi: 10.3389/fpsyt.2021.680811 34149484 PMC8206497

[56] Worsley KJ, Marrett S, Neelin P, Vandal AC, Friston KJ, Evans AC. A unified statistical approach for determining significant signals in images of cerebral activation. Hum Brain Mapp. 1996;4(1):58–73. doi: 10.1002/(SICI)1097-0193(1996)4:1<58::AID-HBM4>3.0.CO;2-O 20408186

[57] Kiebel SJ, Friston KJ. Statistical parametric mapping for event-related potentials: I. Generic considerations. Neuroimage. 2004;22(2):492–502. doi: 10.1016/j.neuroimage.2004.02.012 15193578

[58] Penny WD, Friston KJ, Ashburner JT, Kiebel SJ, Nichols TE. Statistical parametric mapping: the analysis of functional brain images [Internet]. Elsevier; 2011 [cited 2026 Jul 17]. Available from: https://books.google.com/books?hl=it&lr=&id=G_qdEsDlkp0C&oi=fnd&pg=PP1&dq=Random+effects+analysis.+Statistical+parametric+mapping:+The+analysis+of+functional+brain+images&ots=Xo4KABP8YH&sig=VlYL_zHPRtF8Usq5Ol9pDWABeZY

[59] Kilner JM, Friston KJ. Topological inference for EEG and MEG. Ann Appl Stat. 2010;1272–90.

[60] Flandin G, Friston KJ. Analysis of family-wise error rates in statistical parametric mapping using random field theory. Hum Brain Mapp. 2019;40(7):2052–4. doi: 10.1002/hbm.23839 29091338 PMC6585687

[61] Oostenveld R, Fries P, Maris E, Schoffelen J-M. FieldTrip: Open source software for advanced analysis of MEG, EEG, and invasive electrophysiological data. Comput Intell Neurosci. 2011;2011:156869. doi: 10.1155/2011/156869 21253357 PMC3021840

[62] Maris E, Oostenveld R. Nonparametric statistical testing of EEG- and MEG-data. J Neurosci Methods. 2007;164(1):177–90. doi: 10.1016/j.jneumeth.2007.03.024 17517438

[63] Wickham H, François R, Henry L, Müller K, Vaughan D, Software P, et al. dplyr: A Grammar of Data Manipulation [Internet]. 2026 [cited 2026 Jul 17]. Available from: https://cran.r-project.org/web/packages/dplyr/index.html

[64] Wickham H, Vaughan D, Girlich M, Ushey K, Software P, PBC. tidyr: Tidy Messy Data [Internet]. 2025 [cited 2026 Jul 17]. Available from: https://cran.r-project.org/web/packages/tidyr/index.html

[65] Wickham H, Software P, PBC. stringr: Simple, Consistent Wrappers for Common String Operations [Internet]. 2025 [cited 2026 Jul 17]. Available from: https://cran.r-project.org/web/packages/stringr/index.html

[66] Bar M. Predictions: a universal principle in the operation of the human brain. Philos Trans R Soc B Biol Sci. 2009;364(1521):1181. 10.1098/rstb.2008.0321 PMC2666718 19527998

[67] Li H, Ji L, Tong K, Ren N, Chen W, Liu CH, et al. Processing of Individual Items during Ensemble Coding of Facial Expressions. Front Psychol. 2016;7:1332. doi: 10.3389/fpsyg.2016.01332 27656154 PMC5013048

[68] Lee TS, Mumford D. Hierarchical Bayesian inference in the visual cortex. J Opt Soc Am A Opt Image Sci Vis. 2003;20(7):1434–48. doi: 10.1364/josaa.20.001434 12868647

[69] Perry CJ, Fallah M. Feature integration and object representations along the dorsal stream visual hierarchy. Front Comput Neurosci. 2014;8:84. doi: 10.3389/fncom.2014.00084 25140147 PMC4122209

[70] Weilnhammer VA, Stuke H, Sterzer P, Schmack K. The Neural Correlates of Hierarchical Predictions for Perceptual Decisions. J Neurosci. 2018;38(21):5008–21. doi: 10.1523/JNEUROSCI.2901-17.2018 29712780 PMC6596123

[71] Wolpert DM, Doya K, Kawato M. A unifying computational framework for motor control and social interaction. Philos Trans R Soc Lond B Biol Sci. 2003;358(1431):593–602. doi: 10.1098/rstb.2002.1238 12689384 PMC1693134

[72] Kaufman J, Johnston PJ. Facial motion engages predictive visual mechanisms. PLoS One. 2014;9(3):e91038. doi: 10.1371/journal.pone.0091038 24632821 PMC3954613

[73] Genschow O, Brass M. The predictive chameleon: evidence for anticipated social action. J Exp Psychol Hum Percept Perform. 2015;41(2):265–8. doi: 10.1037/xhp0000035 25665086

[74] Chang Y, Xu J, Shi N, Zhang B, Zhao L. Dysfunction of processing task-irrelevant emotional faces in major depressive disorder patients revealed by expression-related visual MMN. Neurosci Lett. 2010;472(1):33–7. doi: 10.1016/j.neulet.2010.01.050 20117175

[75] Lee KM, Ferreira-Santos F, Satpute AB. Predictive processing models and affective neuroscience. Neurosci Biobehav Rev. 2021;131:211–28. doi: 10.1016/j.neubiorev.2021.09.009 34517035 PMC9074371

[76] Kok P, Rahnev D, Jehee JFM, Lau HC, de Lange FP. Attention reverses the effect of prediction in silencing sensory signals. Cereb Cortex. 2012;22(9):2197–206. doi: 10.1093/cercor/bhr310 22047964

[77] Costa C, Pezzetta R, Toffalini E, Grassi M, Cona G, Miniussi C, et al. Enhancing the quality and reproducibility of research: Preferred Evaluation of Cognitive and Neuropsychological Studies - The PECANS statement for human studies. Behav Res Methods. 2025;57(7):182. doi: 10.3758/s13428-025-02705-3 40447861 PMC12125112

